# Recurrent Primary Cutaneous Anaplastic Large Cell Lymphoma With Systemic Involvement: A Case Report and Literature Review

**DOI:** 10.7759/cureus.14284

**Published:** 2021-04-04

**Authors:** Elizabeth Philippe, Kellen T Creech, Nicole Cook, Johanna Segura

**Affiliations:** 1 Family Medicine, Community Health of South Florida, Inc, Miami, USA; 2 Internal Medicine, Nova Southeastern University Dr. Kiran C. Patel College of Osteopathic Medicine, Fort Lauderdale, USA; 3 Public Health, Nova Southeastern University Dr. Kiran C. Patel College of Osteopathic Medicine, Fort Lauderdale, USA; 4 Public Health, Broward County Health Department, Fort Lauderdale, USA

**Keywords:** cutaneous t cell lymphoma, clinical dermatology

## Abstract

Primary cutaneous anaplastic large cell lymphoma (PC-ALCL) is a rare, aggressive neoplasm that frequently relapses and requires the use of multiple treatment modalities. PC-ALCL most commonly presents in patients around the age of 60 and clinically manifests as red, single or sometimes grouped nodular lesions in the skin that tend to ulcerate over time. Although cases are limited to the skin, the extracutaneous spread has been occasionally reported. The diagnosis of PC-ALCL is made through excisional biopsy and subsequent immunohistochemical confirmation. Management of PC-ALCL is dependent on the extent of disease, and most patients can be effectively managed with surgical excision and/or radiation. If relapse occurs, systemic therapy including combination chemotherapy is considered. We present the case of a 43-year-old female who presented to an outpatient clinic with multiple suspicious, red, nodular lesions to her left elbow and right upper back. The further evaluation led to the diagnosis of a stage 4E, ALK-negative, CD30-positive PC-ALCL with recurrence after resection. This case highlights the diagnosis and management of PC-ALCL with systemic involvement that did not respond to initial radiotherapy.

## Introduction

Anaplastic large cell lymphoma (ALCL) is a type of non-Hodgkin lymphoma (NHL) and a subtype of T-cell lymphoma [1]. Generally regarded as an aggressive neoplasm in nature, it comprises approximately 2% of adult NHL cases and is a subtype of peripheral T-cell lymphomas that together makes up approximately 15% of all NHL cases [2]. In the United States, the incidence of ALCL is estimated at 0.25 cases per 100,000 people [1]. This neoplasm also follows a bimodal incidence curve and peaks in adolescence and again around 60 years of age [1]. There are four known types of ALCL: anaplastic lymphoma kinase (ALK) positive primary systemic ALCL, ALK-negative systemic ALCL, breast implant-associated ALCL and primary cutaneous ALCL [2]. In focusing on specifically primary cutaneous ALCL (PC-ALCL), the incidence of this subtype of ALCL is rare and difficult to differentiate from the larger group of CD30-positive cutaneous lymphoproliferative disorders. In an analysis of 157 cases of primary, localized CD30-positive cutaneous lymphoproliferative disorders including PC-ALCL over a 30-year period, the median age of diagnosis was 61 years with a male predominance and most commonly seen within the Caucasian population [3]. Although the majority of systemic ALCL cases are associated with a specific translocation involving the ALK gene on chromosome 2p23, PC-ALCL cases are more commonly associated with being CD30-positive approximately 75% of the time [3]. PC-ALCL most commonly presents as single or multiple reddish nodules that initially appear in the skin, in the lymph nodes, or in various organ systems [3]. Although lesions associated with PC-ALCL partially regress approximately 50% of the time, relapses occur in approximately 40% of cases [4]. Biopsy and immunohistochemical evaluation followed by additional staging assessment are needed to accurately diagnose and determine the extent of the disease [5]. We present the rare case of a recurrent ALK-negative, CD30-positive PC-ALCL with systemic involvement that did not respond to local radiotherapy and other systemic treatment modalities were considered.

## Case presentation

**Figure 1:**
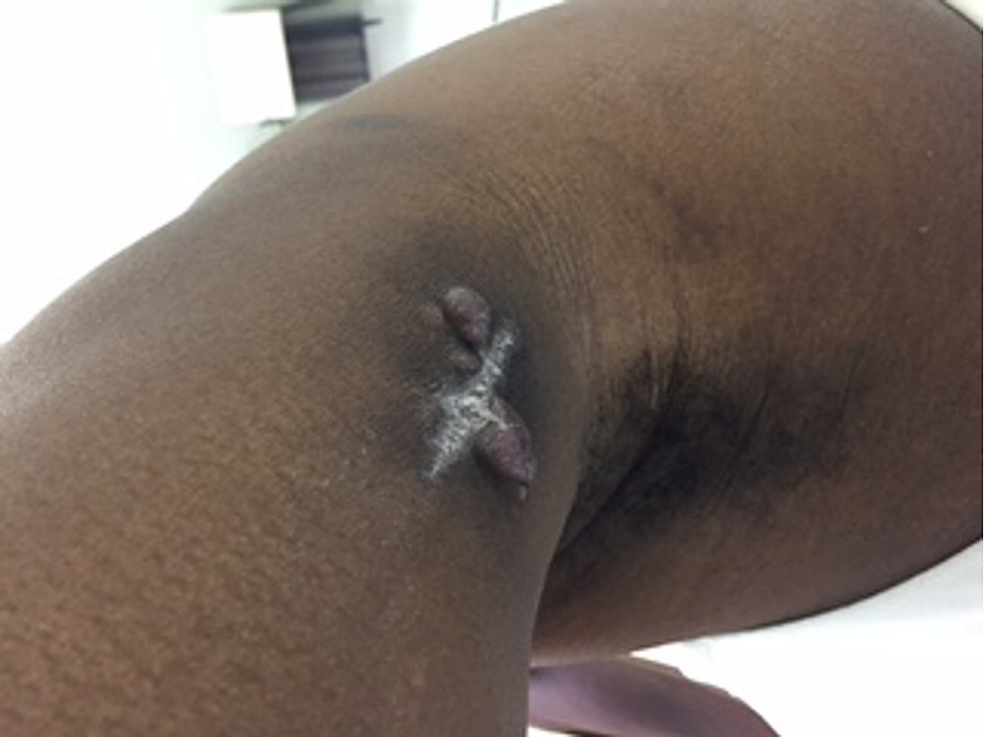
Multiple reddish-brown tumor nodules on the left elbow.

**Figure 2:**
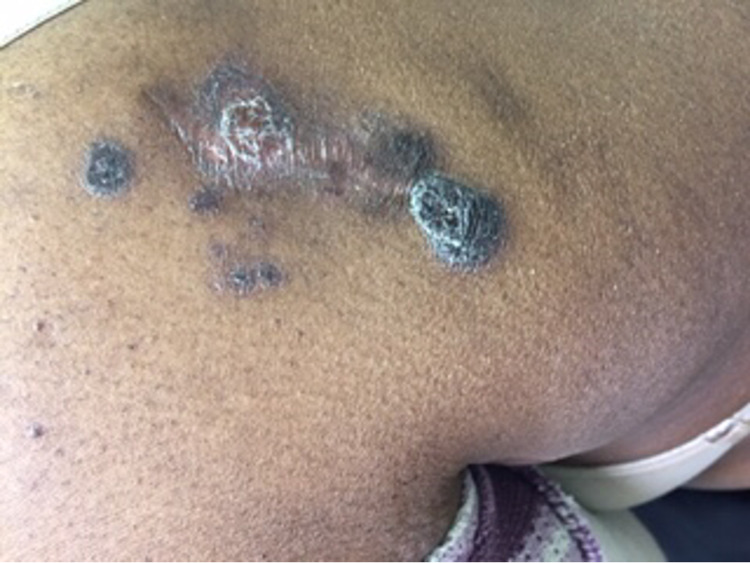
Multiple reddish-brown tumor nodules on the right upper back.

A 43-year-old female presented to an outpatient clinic with swelling of the left lateral elbow, a pink nodular cutaneous lesion on the left elbow (Figure 1) and a right upper back nodular lesion (Figure 2).

**Figure 3:**
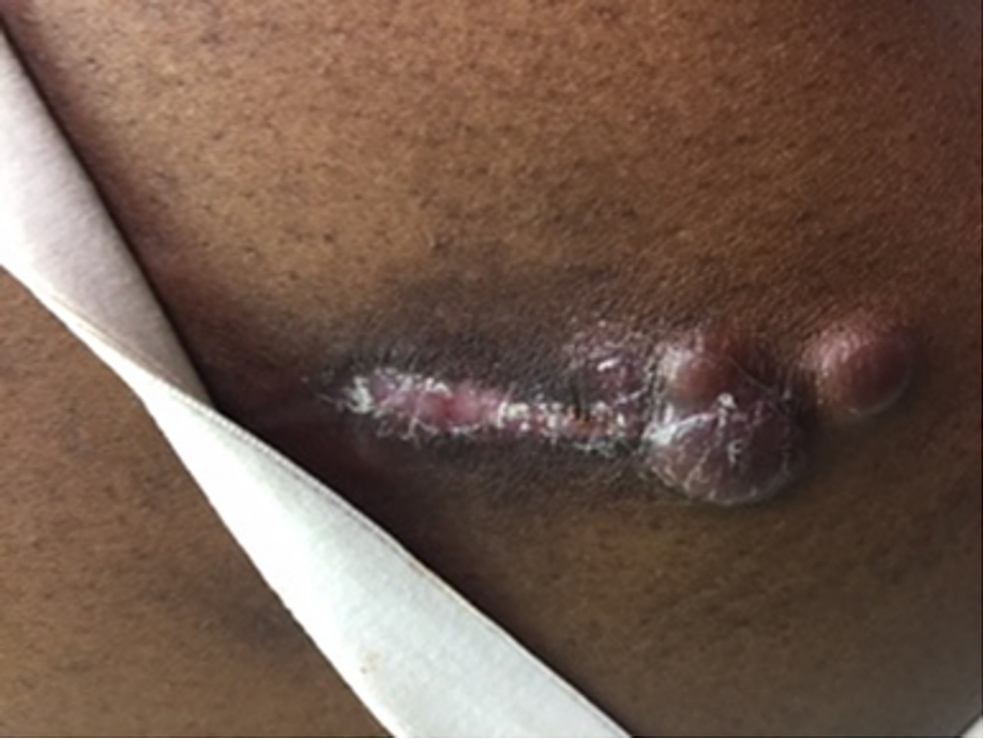
Recurrent pink tumor nodules on left upper arm.

These suspicious nodules prompted further evaluation, and excisional biopsy and imaging were performed. Biopsy and immunophenotyping revealed ALK-negative, CD30-positive primary cutaneous anaplastic large cell lymphoma (PC-ALCL). To further assess the extent of systemic involvement, additional imaging was performed to inspect for the presence of metastasis. Positron emission tomography (PET) and computerized tomography (CT) images showed no focal areas of abnormal fluorodeoxyglucose (FDG) uptake within the skull base or neck. However, these scans detected two FDG avid left axillary lymph nodes, the most prominent left axillary node measured at 1.3 cm x 11.1 cm with a maximum standardized uptake value (SUV max) of 2.44. No suspicious FDG avid pulmonary nodules were appreciated. Intense increased FDG uptake was appreciated within the posterior soft tissues of the left elbow with an SUV max of 8.52, consistent with the patient’s reported history of biopsy-proven lymphoma in this location. Bone marrow involvement was negative, and the CT of the brain revealed no mass effect, no territorial hypodensities, and no acute intracranial process. After staging, the final diagnosis of our patient was a stage 4E, ALK-negative, CD30-positive primary cutaneous ALCL. Local radiotherapy and surgical excision of nodules present on the left arm and upper back was performed. On a follow-up visit, a physical exam revealed the patient had developed recurrent lesions to the left upper arm and right upper back lesions after 20 sessions of radiotherapy (Figure 3). Systemic therapy was then considered and our patient was started on CHOP (cyclophosphamide, doxorubicin, vincristine, and prednisone) therapy for six cycles with growth factor support, in conjunction with radiation therapy to the cutaneous lesions. If the patient should relapse only cutaneously, then an anti-CD30-positive treatment, known as brentuximab, will be offered. Should the patient relapse systemically in the future, then autologous transplantation will be offered.

## Discussion

Primary cutaneous ALCL is a rarely encountered neoplasm with an excellent prognosis (10-year overall survival rate 90%) but a high recurrence rate [3]. It was originally hypothesized that the Epstein-Barr virus may contribute to the development of ALCL, but this has since been refuted [1]. However, infection with the human immunodeficiency virus (HIV) remains a risk factor for ALCL, especially for ALK-negative ALCL [1]. The WHO classifies ALCL into cutaneous and systemic types, and is further stratified by the presence or absence of ALK protein expression [1]. A deeper discussion of the molecular pathogenesis of ALCL details how a similar set of genes are disrupted through different chromosomal translocations. T(2;5), the most commonly observed translocation in ALK-positive ALCL, fuses a segment of the ALK gene on chromosome 2p23 with a portion of the nucleophosmin (NPM1) gene on chromosome 5q35 [1]. This fusion gene encodes for an 80 kDa NPM-ALK chimeric protein with constitutive tyrosine kinase activity [1]. This leads to uninhibited cellular proliferation and the inhibition of apoptosis [1]. Lymphoproliferative disorders of both B-cell and T-cell lineages induced by the expression of NPM-ALK have been corroborated by in-vitro and mouse model studies [1]. In ALK-negative systemic and primary cutaneous ALCL, T(6;7)(p25.3;q32.3) is the translocation present in up to 30% of cases [1]. This translocation leads to reduced expression of DUSP22 (dual specificity phosphatase 22), which encodes a phosphatase that regulates cell signaling and leads to unregulated cell proliferation [1]. Although most cases of PC-ALCL present as isolated lesions that can be effectively managed with radical surgical excision and/or radiation, many of these patients will experience relapse and need additional therapy [3]. A retrospective cohort analysis of 56 patients with CD30-positive cutaneous lymphoproliferative disorders showed that 95% of patients treated with surgical excision achieved complete remission and 41% of patients relapsed within 22 months [3]. Furthermore, 64% of patients treated with surgery in combination with radiation developed the recurrent disease within a 55-month follow-up period [3]. This treatment approach was taken in our case, as our patient was treated initially through surgical excision of lesions and radiation. However, due to the recurrence of lesions, clinicians were led to consider a variety of treatment options. Oral methotrexate is the preferred initial systemic therapy due to its reliable response rate, convenience and reasonable side effect profile [3]. In a small study of 13 patients with PC-ALCL, 10 of these patients responded to treatment with methotrexate within a period of four weeks [3]. Brentuximab, an anti-CD30 monoclonal antibody, is another consideration for patients with PC-ALCL who worsen or become intolerant of primary systemic treatment [2]. Compared with methotrexate, brentuximab proved to be more effective and led to the complete resolution of skin manifestations of PC-ALCL in 10 of 16 patients studied [3]. For patients, like ours, who had increased nodal involvement and failed other treatment modalities, the CHOP (cyclophosphamide, doxorubicin, vincristine, and prednisone) chemotherapy regimen is an appropriate treatment option [3,5]. Although an analysis of 53 patients treated with CHOP gave rise to a 92% complete response rate, 62% of patients exhibited recurrent disease within four months [3]. Finally, allogeneic hematopoietic cell transplantation may be discussed as another treatment option in patients with numerous relapses after treatment for systemic disease [2]. This case adds to the lack of current literature surrounding the management of PC-ALCL and will hopefully encourage the pursuit of an effective therapy for PC-ALCL that will lead to increased rates of complete remission and drastically reduce the reported 40% relapse rate associated with this rare neoplasm [4].

## Conclusions

ALCL is a rarely encountered form of NHL and subtype of peripheral T-cell lymphoma. Primary cutaneous ALCL commonly manifests as painless adenopathy or reddish, nodular lesions that can ulcerate and commonly relapses. Biopsy, immunohistochemical analysis and staging determine the extent of the disease and treatment course. Initial treatment of primary cutaneous ALCL is surgical removal and/or radiation. However, if recurrence or rare systemic involvement is encountered, then agents like methotrexate or chemotherapy are considered. Though the literature and research concerning the management of PC-ALCL with systemic involvement are scarce, this case highlights how initial treatment options proved futile and how important it is to develop new treatment approaches to this disease.
